# The Genetic Landscape of Diabetes Mellitus: Lessons from Monogenic and Polygenic Forms

**DOI:** 10.3390/life16030399

**Published:** 2026-03-01

**Authors:** Davide Nilo, Roberto Nilo, Marta Chiara Sircana, Ferdinando Carlo Sasso, Carlo Acierno, Leonilde Bonfrate, Alfredo Caturano

**Affiliations:** 1 Department of Advanced Medical and Surgical Sciences, University of Campania “Luigi Vanvitelli’’, 80138 Naples, Italy; nilodavide@gmail.com (D.N.); ferdinandocarlo.sasso@unicampania.it (F.C.S.); 2 Meditrial Europe, 00198 Roma, Italy; robertonilo98@gmail.com; 3 Department of Medical, Surgical and Pharmacology, University of Sassari, 07100 Sassari, Italy; m.sircana4@phd.uniss.it; 4 Azienda Ospedaliera Regionale San Carlo, 85100 Potenza, Italy; carlo894@gmail.com; 5 Department of Human Sciences and Promotion of the Quality of Life, San Raffaele Roma University, 00166 Rome, Italy; leonilde.bonfrate@uniroma5.it; 6 Center of Nutrition for the Research and the Care of Obesity and Metabolic Diseases, National Institute of Gastroenterology IRCCS “Saverio de Bellis”, 70013 Castellana Grotte, Italy

**Keywords:** diabetes heterogeneity, monogenic diabetes, polygenic risk, β-cell dysfunction, precision diabetology, genetic continuum

## Abstract

Diabetes mellitus is increasingly recognized as a biologically heterogeneous disorder that extends beyond traditional phenotype-based classifications. Advances in human genetics have revealed that monogenic and polygenic forms of diabetes are not discrete entities, but rather represent points along a continuum of genetic architectures that converge on shared molecular pathways governing pancreatic β-cell identity, function, and survival. Rare monogenic forms, including maturity-onset diabetes of the young and neonatal diabetes, arise from highly penetrant single-gene defects that directly impair transcriptional regulation, glucose sensing, insulin biosynthesis, or stimulus–secretion coupling. Although individually uncommon, these disorders provide high-resolution models of β-cell dysfunction and have demonstrated the clinical value of genotype-guided diagnosis and therapy. At the opposite end of the spectrum, type 1 and type 2 diabetes result from complex interactions between multiple genetic variants and environmental factors, with genome-wide association studies highlighting a central role for genetically determined β-cell vulnerability alongside immune-mediated and metabolic stress pathways. Importantly, intermediate phenotypes such as latent autoimmune diabetes in adults further illustrate the overlap between autoimmune and metabolic mechanisms, challenging rigid diagnostic boundaries. This review synthesizes current evidence on the genetic architecture of diabetes across monogenic and polygenic forms, emphasizing convergent molecular mechanisms and their translational implications. By integrating insights from rare genetic disorders with findings from large-scale population studies, we propose a continuum-based framework that supports a shift from phenotype-driven labels toward a mechanistic, biology-informed approach to diabetes classification, risk stratification, and personalized care.

## 1. Introduction

Diabetes mellitus comprises a heterogeneous group of metabolic disorders characterized by chronic hyperglycemia resulting from defects in insulin secretion, insulin action, or a combination of both [1]. For decades, diabetes has been classified primarily into type 1 diabetes mellitus (T1DM) and type 2 diabetes mellitus (T2DM), a distinction that remains clinically useful but increasingly insufficient to capture the underlying biological diversity of the disease [2]. Accumulating evidence from clinical observation, genetic studies, and molecular profiling indicates that diabetes is better conceptualized as a spectrum of disorders with overlapping phenotypes, distinct pathogenic mechanisms, and variable responses to therapy [3]. In this context, the traditional dichotomy between insulin deficiency and insulin resistance represents a simplification that does not fully reflect the complexity of glucose dysregulation in humans.

From an epidemiological perspective, diabetes represents a major and growing global health burden. T2DM accounts for the vast majority of cases worldwide and continues to rise in prevalence, driven by population aging, increasing rates of obesity, sedentary lifestyles, and adverse dietary patterns [4]. Nevertheless, even within T2DM, there is marked heterogeneity in age at onset, tempo of disease progression, degree of insulin resistance, residual β-cell function, and susceptibility to complications [5]. Similarly, T1DM, classically viewed as a homogeneous autoimmune disorder, exhibits substantial variability in terms of age at diagnosis, intensity of immune-mediated β-cell destruction, persistence of endogenous insulin secretion, and clinical course [6]. These observations challenge purely phenotype-based classifications and suggest that shared clinical labels may mask fundamentally different biological processes [7].

Over the past two decades, advances in human genetics have profoundly reshaped our understanding of diabetes pathophysiology. Rather than representing discrete entities, the major forms of diabetes appear to lie along a genetic continuum, ranging from rare, highly penetrant monogenic disorders to common polygenic diseases influenced by hundreds of genetic variants with small individual effects [8,9]. At one end of this continuum, monogenic diabetes, including maturity-onset diabetes of the young (MODY) and neonatal diabetes, results from pathogenic variants in single genes that directly impair pancreatic β-cell development, glucose sensing, or insulin secretion. Although individually rare, these conditions provide uniquely informative models of β-cell biology, allowing precise dissection of gene–function–phenotype relationships and offering clear examples of genotype-guided therapy [10,11].

At the opposite end of the spectrum, T1DM and T2DM arise from complex interactions between multiple genetic loci and environmental exposures [3]. In T1DM, genetic susceptibility is dominated by variants within the human leukocyte antigen (HLA) region, with additional contributions from non-HLA loci involved in immune regulation, tolerance, and antiviral responses [12]. In T2DM, genome-wide association studies have identified hundreds of risk loci, most of which reside in non-coding regions and exert regulatory effects on gene expression rather than altering protein structure [13]. Of note, many of these variants converge on pathways related to β-cell function, survival, and stress responses, shifting the pathogenic emphasis from insulin resistance alone toward intrinsic β-cell vulnerability [14]. Importantly, genetic predisposition does not operate in isolation. Environmental factors such as obesity, physical inactivity, dietary composition, intrauterine exposures, and early-life metabolic programming strongly modulate the phenotypic expression of genetic risk [15]. This gene–environment interplay helps explain why individuals with similar genetic backgrounds may follow divergent clinical trajectories and why diabetes phenotypes can evolve over time [16]. Moreover, emerging evidence suggests that epigenetic mechanisms and metabolic memory further shape disease progression, linking past exposures to future β-cell dysfunction and metabolic deterioration [17].

The clinical implications of this evolving genetic landscape are substantial. Misclassification of diabetes subtype remains common, particularly in young adults, lean individuals, and patients with atypical clinical features. In such cases, failure to recognize a monogenic etiology can lead to inappropriate treatment, unnecessary insulin use, and missed opportunities for family screening [18]. Conversely, improved understanding of polygenic risk has opened new avenues for risk stratification, subclassification of diabetes, and identification of individuals predisposed to early-onset disease or rapid β-cell failure [19,20]. While the clinical application of polygenic risk scores (PRS) remains limited by ancestry bias and environmental modulation, their conceptual contribution to redefining diabetes heterogeneity is undeniable [21]. Against this background, diabetes increasingly emerges not as a collection of rigid diagnostic categories, but as a continuum of disorders unified by shared molecular pathways affecting β-cell identity, metabolism, and survival [22]. Monogenic and polygenic forms, once considered distinct, are now recognized to converge on overlapping biological networks involving transcriptional regulation, mitochondrial function, endoplasmic reticulum stress, and insulin biosynthesis [23]. Appreciating these commonalities offers an opportunity to move beyond symptom-based classification toward a mechanistic framework that better aligns diagnosis with pathophysiology.

The aim of this review is therefore to synthesize current knowledge on the genetic architecture of diabetes across monogenic and polygenic forms, highlighting shared molecular mechanisms and translational implications. By integrating insights from rare genetic disorders with findings from large-scale population studies, we seek to illustrate how genetics can inform a more precise, biologically grounded approach to diabetes classification, diagnosis, and personalized care.

## 2. Monogenic Diabetes: Models of Primary β-Cell Dysfunction

### 2.1. Overview

Monogenic diabetes encompasses a heterogeneous group of disorders caused by pathogenic variants in a single gene that directly impair pancreatic β-cell development, glucose sensing, insulin biosynthesis, or stimulus–secretion coupling [24]. In contrast to polygenic diabetes, which arises from the cumulative effects of numerous common variants interacting with environmental exposures, monogenic forms are driven by rare, high-impact mutations that confer relatively strong and often predictable genotype–phenotype relationships [25]. Inheritance is most commonly autosomal dominant, particularly in MODY, although autosomal recessive inheritance and de novo variants are well described, especially in neonatal diabetes and syndromic presentations [26]. Despite their relatively low prevalence, monogenic forms of diabetes are of disproportionate clinical and biological importance. Clinically, they account for a meaningful fraction of diabetes cases diagnosed at a young age and are frequently misclassified as type 1 or type 2 diabetes, leading to inappropriate treatment strategies and missed opportunities for targeted therapy [27]. Biologically, monogenic diabetes offers a unique window into the fundamental mechanisms governing β-cell identity, insulin secretion, and cellular resilience. Because single-gene defects often disrupt key regulatory nodes, these conditions function as natural “human models” that reveal pathways later found to be more subtly perturbed in common polygenic diabetes [28].

From a phenotypic perspective, individuals with monogenic diabetes often share features that distinguish them from classical T1DM or T2DM, including early onset of hyperglycemia, absence of pancreatic autoimmunity, and preserved endogenous insulin secretion, commonly reflected by measurable C-peptide levels well beyond the timeframe expected for autoimmune β-cell destruction. A non-obese or only mildly overweight phenotype and a multigenerational family history consistent with autosomal dominant inheritance are frequent but not universal findings [29]. Importantly, penetrance is variable, and contemporary environmental factors, such as obesity and insulin resistance, can modify clinical expression, leading to phenotypic overlap with type 2 diabetes, particularly in adulthood [30].

Within this framework, MODY and neonatal diabetes represent complementary models of monogenic β-cell dysfunction. MODY predominantly illustrates progressive defects in transcriptional regulation and glucose sensing, whereas neonatal diabetes highlights early-life disruption of insulin secretion and cellular stress responses. Together, these entities form the conceptual backbone for understanding how discrete genetic lesions translate into diverse clinical phenotypes [29].

### 2.2. Maturity-Onset Diabetes of the Young (MODY)

**Figure 1:**
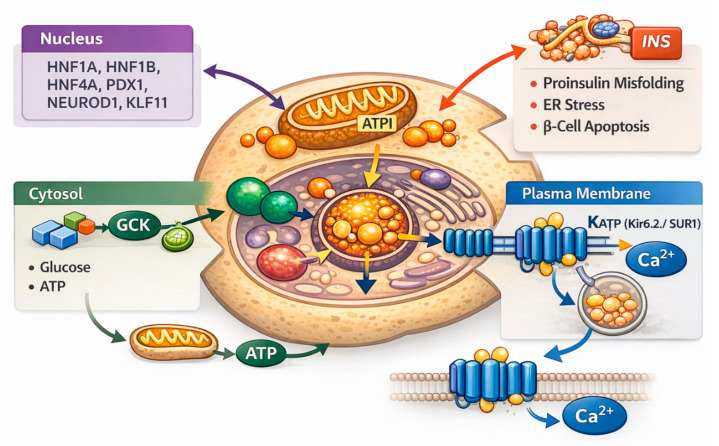
Molecular mechanisms of β-cell dysfunction in MODY, emphasizing convergent pathways shared with other monogenic diabetes subtypes. Pathogenic variants in MODY-associated genes disrupt distinct but converging components of glucose-stimulated insulin secretion in pancreatic β-cells. Mutations in key transcription factors (*HNF1A*, *HNF4A*, *HNF1B*, *PDX1*, *NEUROD1*, *KLF11*) impair β-cell identity, differentiation, and insulin gene transcription. Glucokinase (*GCK*) variants alter intracellular glucose sensing and ATP generation, increasing the glycemic threshold required to trigger insulin secretion. Variants in the insulin gene (INS) promote proinsulin misfolding, endoplasmic reticulum stress, and progressive β-cell dysfunction. For mechanistic completeness, dysfunction of the ATP-sensitive potassium (KATP) channel (Kir6.2/SUR1), which primarily underlies neonatal diabetes but may contribute to MODY-like phenotypes in selected contexts, is also shown, highlighting impaired membrane depolarization and calcium-dependent insulin exocytosis. Together, these mechanisms illustrate how single-gene defects converge on primary β-cell dysfunction, producing heterogeneous clinical phenotypes and genotype-specific therapeutic responses.

Maturity-onset diabetes of the young represents the prototypical autosomal dominant form of monogenic diabetes and serves as a canonical model of primary β-cell dysfunction (Figure 1). Rather than a single disease entity, MODY encompasses a spectrum of genetic disorders unified by impaired insulin secretion in the absence of autoimmunity, but distinguished by the specific molecular pathways affected. The majority of MODY genes encode proteins involved in β-cell transcriptional regulation or glucose sensing, underscoring the central role of these processes in maintaining glucose homeostasis [10,26].

The most frequent and clinically actionable MODY subtypes involve transcription factors that orchestrate β-cell gene expression and differentiation. *HNF1A* (MODY3) encodes hepatocyte nuclear factor-1α, a master regulator of transcriptional programs governing glucose transport, glycolysis, and insulin secretion; pathogenic variants typically result in progressive β-cell dysfunction with age [31,32]. *HNF4A* (MODY1) encodes hepatocyte nuclear factor-4α, which operates upstream of *HNF1A*-related networks and influences both β-cell and hepatic metabolic pathways [33,34,35]. *HNF1B* (MODY5) plays a broader developmental role, and its disruption is frequently associated with extra-pancreatic manifestations, particularly renal and urogenital abnormalities, reflecting its involvement in organogenesis [36,37]. A distinct mechanistic paradigm is provided by *GCK* (MODY2), which encodes glucokinase, the β-cell’s primary glucose sensor. Heterozygous loss-of-function variants raise the threshold for glucose-stimulated insulin secretion, leading to stable, mild fasting hyperglycemia with limited postprandial excursions and a generally benign clinical course. This form of MODY exemplifies how altered glucose sensing, rather than progressive β-cell failure, can underlie dysglycemia [38,39]. Rarer MODY subtypes further illuminate key aspects of β-cell biology. Variants in *PDX1* and *NEUROD1* impair pancreatic development and β-cell differentiation, occasionally resulting in reduced pancreatic mass or variable degrees of insulin deficiency [40,41]. KLF11 influences insulin gene transcription and β-cell survival pathways, while CEL mutations may affect exocrine–endocrine interactions and pancreatic integrity [42,43]. Pathogenic variants in INS, the insulin gene itself, disrupt proinsulin folding and processing, inducing β-cell stress and producing phenotypes that range from MODY-like diabetes to neonatal diabetes depending on the nature of the mutation [44,45]. Similarly, although dysfunction of the ATP-sensitive potassium (KATP) channel is classically associated with neonatal diabetes, heterozygous variants, particularly in *ABCC8*, have been reported in MODY-like phenotypes with later onset, highlighting mechanistic overlap across monogenic diabetes subtypes [46].

Although clinical features may overlap across MODY subtypes, the underlying molecular defect strongly influences disease trajectory and therapeutic responsiveness. Individuals with *HNF1A*- or *HNF4A*-related diabetes typically exhibit marked sensitivity to sulfonylureas, reflecting preserved β-cell mass with impaired glucose-stimulated secretion; low-dose sulfonylureas can often replace insulin therapy when misclassification has occurred [47]. In contrast, *GCK*-MODY generally does not require pharmacological treatment outside specific contexts, such as pregnancy, because hyperglycemia is mild, non-progressive, and poorly responsive to glucose-lowering agents [48].

Genetic confirmation of MODY has immediate and lasting clinical implications. It enables accurate classification, avoidance of unnecessary insulin therapy, tailored follow-up for associated comorbidities, and cascade testing of family members. Importantly, the diagnostic yield of genetic testing is highest when applied to carefully selected individuals with young-onset diabetes, preserved C-peptide, negative islet autoantibodies, and a family history compatible with dominant inheritance, while acknowledging variable penetrance and environmental modulation.

### 2.3. Neonatal Diabetes Mellitus (NDM)

Neonatal diabetes mellitus is defined by diabetes onset before six months of age, a clinically critical threshold given the extreme rarity of autoimmune T1DM in this period [49]. As such, neonatal diabetes is overwhelmingly monogenic in origin and may present as either transient or permanent disease. It can occur as an isolated endocrine disorder or as part of a broader syndromic phenotype [50].

Neonatal diabetes represents the clearest proof-of-concept for precision medicine in diabetology, as molecular diagnosis frequently leads to fundamental changes in treatment and prognosis. The most common genetic causes involve disruption of stimulus–secretion coupling and β-cell survival [11]. Activating variants in *KCNJ11* and *ABCC8*, encoding the Kir6.2 and SUR1 subunits of the β-cell ATP-sensitive potassium (KATP) channel, prevent membrane depolarization and calcium influx, thereby suppressing insulin secretion [51]. These mutations exemplify how altered β-cell excitability can produce severe hyperglycemia from birth.

Other causes of neonatal diabetes include pathogenic variants in INS, which impair insulin biosynthesis and induce endoplasmic reticulum stress, and in *GCK*, particularly in biallelic or functionally severe contexts, resulting in profound defects in glucose sensing [52,53]. Variants in *EIF2AK3* (PERK) disrupt the unfolded protein response and render β-cells highly vulnerable to secretory stress, often within multisystem syndromes [54].

The therapeutic implications of genetic diagnosis in neonatal diabetes are profound. Many individuals with *KCNJ11*- or *ABCC8*-related diabetes can successfully transition from insulin to oral sulfonylureas, which close KATP channels pharmacologically and restore insulin secretion in a substantial proportion of cases [46,55]. Early identification is therefore critical, as it can dramatically reduce treatment burden and, in some cases, improve neurodevelopmental outcomes. Even when insulin therapy remains necessary, molecular diagnosis informs prognosis, guides surveillance for associated features, and enables family counseling.

### 2.4. Lessons from Monogenic Forms

Beyond their direct diagnostic relevance, monogenic forms of diabetes act as magnifying lenses through which the core mechanisms of β-cell failure can be observed with exceptional clarity [29]. Importantly, the pathways disrupted in monogenic diabetes, such as transcriptional dysregulation, impaired glucose sensing, defective stimulus–secretion coupling, mitochondrial dysfunction, and maladaptive cellular stress responses, are not unique to rare disorders. Rather, they represent shared biological vulnerabilities that are more subtly and cumulatively perturbed in polygenic diabetes [56]. In this perspective, monogenic diabetes provides a conceptual foundation for understanding diabetes as a biologically continuous disorder, rather than a collection of discrete categories. Insights gained from single-gene defects have directly informed models of β-cell dysfunction in T1DM and T2DM, reinforcing the idea that diverse genetic architectures converge on common molecular networks. Consequently, monogenic diabetes should not be viewed merely as a set of rare diagnoses to be identified and managed but as a critical framework for redefining diabetes classification, refining risk stratification, and advancing the development of personalized therapeutic strategies (Table 1).

## 3. Polygenic Diabetes

### 3.1. Type 1 Diabetes Mellitus (Autoimmune)

T1DM is a chronic autoimmune disease characterized by immune-mediated destruction of pancreatic β-cells, resulting in absolute insulin deficiency and lifelong dependence on exogenous insulin therapy [57]. Among common forms of diabetes, T1DM exhibits one of the highest heritability estimates, underscoring the central role of genetic susceptibility, while also requiring environmental exposures to trigger disease onset [57,58].

The strongest genetic determinants of T1DM reside within the human leukocyte antigen (HLA) class II region on chromosome 6p21, where specific HLA-DR and HLA-DQ haplotypes influence antigen presentation, thymic T-cell selection, and peripheral immune tolerance [58]. These alleles critically shape the autoreactive T-cell repertoire directed against β-cell antigens and account for a substantial proportion of inherited disease risk [58,59]. Importantly, HLA-associated risk is neither deterministic nor uniform, contributing to the observed variability in age at onset and disease progression.

Beyond the HLA region, genome-wide association studies have identified more than 60 non-HLA loci associated with T1DM susceptibility [59,60]. Notably, the majority of these loci encode proteins involved in immune regulation rather than intrinsic β-cell biology, including pathways governing T-cell activation, immune checkpoint signaling, cytokine responses, and regulatory T-cell homeostasis [59,60,61]. Variants affecting insulin gene expression further contribute to loss of immune tolerance by altering antigen availability during thymic education [59,61].

Innate immune mechanisms also play a role in disease initiation, with genetic variation in antiviral response genes linking viral exposure to the development of islet autoimmunity [59,62]. Environmental modifiers, such as early-life microbiome composition, nutritional factors, and vitamin D status, interact with genetic susceptibility to modulate disease penetrance, timing of onset, and progression [58,62]. Together, these findings support a model in which T1DM arises from a genetically primed immune system that, upon environmental challenge, targets β-cells through sustained autoimmune inflammation

### 3.2. Type 2 Diabetes Mellitus (Metabolic)

T2DM is a highly prevalent multifactorial metabolic disorder characterized by progressive β-cell dysfunction developing on a background of variable insulin resistance [61,63]. Although traditionally viewed as a consequence of obesity and lifestyle factors, genetic studies have demonstrated that inherited susceptibility plays a substantial role in determining individual risk, age at onset, and disease trajectory [62,63,64].

Large-scale genome-wide association studies have identified hundreds of loci associated with T2DM risk, collectively explaining a significant proportion of disease heritability [62,63,64]. Strikingly, most T2DM-associated variants map to non-coding regions of the genome and exert regulatory effects on gene expression rather than altering protein structure [62,64]. Functional studies have shown that many of these variants act within pancreatic islets, influencing transcriptional programs that govern insulin secretion, β-cell survival, and adaptive responses to metabolic stress [62,63,64,65].

Among the strongest and most consistently replicated associations, variants in TCF7L2 confer the highest genetic risk for T2DM by impairing insulin secretion, incretin signaling, and proinsulin processing [63,65]. Additional loci implicate pathways involved in adipogenesis, insulin signaling, zinc transport, mitochondrial function, and β-cell stress responses, reflecting the biological heterogeneity underlying T2DM susceptibility [62,63,64,65,66]. These findings have shifted the pathogenic paradigm from an exclusive focus on insulin resistance toward a model in which genetically determined β-cell vulnerability plays a central role.

Importantly, genetic predisposition to T2DM is strongly modulated by environmental exposures, including excess adiposity, sedentary behavior, and dietary composition [66]. Gene–environment interactions help explain why individuals with similar lifestyles may exhibit markedly different metabolic outcomes and why β-cell failure occurs early in some individuals but only after prolonged insulin resistance in others. This interaction further reinforces the concept of T2DM as a spectrum of biologically distinct subtypes rather than a single homogeneous disease.

### 3.3. Polygenic Risk and Prediction

PRS integrate the cumulative effects of multiple common genetic variants into a single quantitative estimate of inherited susceptibility to disease [67,68,69]. In T1DM, PRS incorporating both HLA and non-HLA loci have demonstrated the ability to identify individuals at increased risk prior to clinical onset and may support stratification for prevention strategies or immunomodulatory trials [58,60,67].

In T2DM, PRS exhibit moderate predictive performance at the individual level but appear particularly informative for identifying individuals predisposed to early-onset diabetes, rapid metabolic deterioration, or predominant β-cell dysfunction [68,69]. Importantly, individuals in the highest percentiles of polygenic risk may reach a lifetime risk comparable to that observed in certain monogenic forms of diabetes [69]. However, the clinical utility of PRS is currently limited by ancestry-specific performance, incomplete representation of diverse populations, and substantial modulation by environmental and lifestyle factors [68,70] (Table 2).

Despite these limitations, PRS represent valuable tools for dissecting diabetes heterogeneity and for advancing research into precision prevention and subclassification. When integrated with clinical, metabolic, and behavioral data, polygenic risk profiling may contribute to identifying biologically defined diabetes subtypes and to tailoring preventive interventions, even if routine clinical implementation remains premature [67,68,69].

## 4. Molecular and Pathophysiological Overlaps

### 4.1. Shared Molecular Pathways in Monogenic and Polygenic Diabetes

Although monogenic and polygenic forms of diabetes differ markedly in inheritance patterns, allele frequencies, and penetrance, they converge on a limited set of shared molecular pathways governing β-cell identity, function, and survival. This convergence challenges traditional distinctions between “rare” and “common” diabetes and supports the view that diverse genetic architectures ultimately disrupt the same biological systems [65,71]. Rather than representing separate disease entities, monogenic and polygenic diabetes can therefore be understood as points along a continuum of β-cell vulnerability.

One of the clearest areas of overlap involves β-cell transcriptional regulation. Transcription factors implicated in monogenic diabetes, such as *HNF1A*, *HNF4A*, *PDX1*, and *NEUROD1*, are not only essential for β-cell development and maintenance, but also act as regulatory hubs within gene networks affected by common T2DM risk variants [71,72]. Genome-wide association studies have shown that many polygenic risk loci map to enhancers and promoters active in pancreatic islets, influencing the expression of genes that control insulin secretion and β-cell adaptive capacity [65,72]. Thus, monogenic defects and polygenic regulatory variation converge on the same transcriptional circuits, differing primarily in the magnitude rather than the nature of their effects.

Mitochondrial metabolism represents another shared vulnerability across the diabetes spectrum. Efficient ATP production is essential for glucose-stimulated insulin secretion, linking cellular metabolism directly to membrane depolarization and insulin exocytosis [65,73]. In monogenic diabetes, mutations affecting glucose sensing or mitochondrial function can produce early and pronounced defects in insulin secretion. In polygenic diabetes, cumulative genetic and environmental insults progressively impair mitochondrial oxidative phosphorylation, increase reactive oxygen species production, and reduce metabolic flexibility [73,74]. These changes contribute to β-cell dysfunction and loss, particularly under conditions of chronic nutrient excess.

Closely related to mitochondrial impairment is the role of oxidative stress, which acts both as a mediator and amplifier of β-cell failure. β-cells are intrinsically vulnerable to oxidative damage due to relatively low antioxidant capacity. Genetic variants that affect mitochondrial function, redox balance, or stress response pathways can therefore predispose β-cells to injury across both rare and common forms of diabetes [73,74]. Over time, oxidative stress not only impairs insulin secretion but also promotes inflammatory signaling and apoptotic pathways.

A further unifying mechanism is endoplasmic reticulum (ER) stress and maladaptive activation of the unfolded protein response (UPR). Proper folding and processing of proinsulin place a substantial burden on the ER, particularly in states of increased insulin demand [65,74]. In monogenic diabetes, pathogenic variants in genes such as *INS* or *EIF2AK3* directly disrupt protein folding or ER stress signaling, leading to early β-cell failure [74,75]. In polygenic diabetes, chronic metabolic stress, lipotoxicity, and inflammatory signaling progressively overwhelm ER homeostasis, resulting in impaired proinsulin processing, reduced insulin secretion, and eventual β-cell apoptosis [74,75]. These observations reinforce the concept that ER stress is not an isolated feature of rare syndromes, but a central pathway in β-cell demise.

Importantly, the convergence of these molecular mechanisms generates overlapping clinical phenotypes that defy simple categorical classification.

Latent autoimmune diabetes in adults (LADA) further exemplifies this continuum-based model. Clinically positioned between classical type 1 and type 2 diabetes, LADA combines features of autoimmune β-cell destruction with a slower rate of progression and variable degrees of insulin resistance [76,77,78]. Genetic studies indicate that LADA shares susceptibility loci with type 1 diabetes, particularly within immune-related pathways, while also displaying overlap with type 2 diabetes risk variants linked to β-cell function [79,80]. As such, LADA reinforces the concept that diabetes phenotypes reflect differing combinations and temporal dynamics of immune-mediated injury and intrinsic β-cell vulnerability rather than discrete disease entities. In this context, LADA represents a clinically relevant example of how immune-mediated and intrinsic β-cell stress pathways may coexist and interact within a single disease trajectory.

Individuals with monogenic diabetes may present later in life with features resembling T2DM, particularly when obesity or insulin resistance coexist [28,81]. Conversely, subsets of patients diagnosed with T2DM may exhibit predominant β-cell failure driven by genetic susceptibility rather than marked insulin resistance, aligning more closely with monogenic or MODY-like pathophysiology [28]. Such phenotypic overlap has been corroborated by data-driven clustering approaches, which identify biologically distinct diabetes subgroups with differing genetic signatures, disease progression, and complication risk [28].

Taken together, these shared molecular and clinical features support a continuum-based model of diabetes pathophysiology, in which diverse genetic perturbations converge on common β-cell stress pathways. Within this framework, monogenic diabetes represents an extreme but informative manifestation of mechanisms that operate more subtly and cumulatively in polygenic disease. Recognizing these overlaps not only enhances biological understanding, but also provides a rationale for rethinking diabetes classification, moving from phenotype-driven labels toward a mechanistic taxonomy grounded in molecular vulnerability and functional impairment.

### 4.2. Experimental Models of Monogenic and Polygenic Diabetes: Translational Insights and Limitations

To complement human genetic evidence, experimental models have played a central role in functionally validating monogenic and polygenic mechanisms of diabetes. By simplifying genetic architecture within controlled environments, these systems allow precise mechanistic dissection of β-cell dysfunction, immune dysregulation, and metabolic stress. At the same time, their inherent reductionism underscores the complexity of the human condition, highlighting both the strengths and translational limitations of modeling diabetes along a genetic continuum [23].

#### 4.2.1. Monogenic Experimental Models: Mechanistic Resolution and Generalizability Constraints

Monogenic murine models exemplify how single-gene perturbations can recapitulate defined pathogenic pathways. Among the most extensively studied is the db/db mouse, which carries a loss-of-function mutation in the leptin receptor (*Lepr*). This defect results in severe obesity, insulin resistance, hyperphagia, and progressive β-cell failure [82,83]. The db/db model has been instrumental in elucidating the role of leptin signaling in energy homeostasis, the relationship between adiposity and insulin resistance, and the dynamics of β-cell compensation under sustained metabolic stress. Its reproducibility and clear genotype–phenotype relationship make it a powerful platform for metabolic investigation and pharmacological testing [84,85,86].

However, the translational scope of the db/db model is inherently constrained. Human T2DM is rarely driven by single-gene defects in leptin signaling and instead reflects polygenic susceptibility interacting with environmental exposures. The db/db phenotype is extreme and rapidly progressive, with uniform expression across inbred strains, contrasting with the gradual and heterogeneous progression observed in humans [86]. Environmental variability, a major determinant of human T2D risk, is minimal under laboratory conditions [87]. Thus, while monogenic models such as db/db mice illuminate discrete metabolic pathways, they do not fully capture the polygenic, environmentally modulated nature of common diabetes.

Other monogenic models further clarify specific mechanisms of β-cell vulnerability. The Akita mouse, harboring a mutation in *Ins2*, develops diabetes secondary to proinsulin misfolding and endoplasmic reticulum stress [88]. This model has provided critical insights into unfolded protein response activation, oxidative stress, and stress-induced β-cell apoptosis [88,89]. Yet again, disease penetrance and severity exceed typical human presentations, reinforcing that monogenic experimental systems represent amplified versions of mechanisms that operate more subtly in polygenic disease.

#### 4.2.2. Polygenic Experimental Models: Immune Complexity and Translational Gaps

In contrast to monogenic systems, polygenic models attempt to approximate the multifactorial architecture of autoimmune diabetes. The non-obese diabetic (NOD) mouse is the prototypical polygenic model of T1DM [90]. Spontaneous diabetes in NOD mice arises from multiple susceptibility loci, including major histocompatibility complex (MHC) variants that influence antigen presentation and immune tolerance [91,92]. This model has been foundational in defining T-cell-mediated β-cell destruction, regulatory T-cell dysfunction, cytokine-driven inflammation, and the staged progression of islet autoimmunity [93,94].

The NOD mouse has also served as a principal platform for preclinical immunomodulatory strategies [95]. Numerous interventions targeting costimulatory pathways, cytokines, or immune checkpoints demonstrated efficacy in preventing or delaying diabetes in this model [96,97,98]. However, translation to human T1DM has often proven challenging. Disease penetrance in female NOD mice approaches near universality under specific housing conditions, whereas human T1DM exhibits incomplete penetrance and marked heterogeneity in age at onset and progression. The accelerated disease course, genetic homogeneity of inbred strains, and strong microbiome sensitivity differ substantially from the diverse and dynamic exposures encountered in human populations [95,99,100].

These discrepancies highlight a central paradox: polygenic murine models capture essential immunological mechanisms yet still simplify the genetic and environmental diversity characteristic of human T1DM. Consequently, therapeutic successes in NOD mice have not consistently translated into durable clinical benefit in humans, underscoring the gap between mechanistic validity and clinical predictability.

#### 4.2.3. Additional Experimental Systems Along the Genetic Spectrum

Beyond the db/db and NOD paradigms, additional models further illustrate the spectrum of experimental diabetes research. The ob/ob mouse, characterized by leptin deficiency, clarified the endocrine regulation of appetite and adiposity, reinforcing the hormonal axis linking obesity to insulin resistance [101,102]. The New Zealand Obese (NZO) mouse, a polygenic model of obesity-associated diabetes, more closely approximates multifactorial T2D by combining inherited susceptibility with diet-induced metabolic stress [103]. In contrast, chemically induced models such as streptozotocin (STZ) exposure produce β-cell ablation without genetic predisposition, offering experimental control but lacking immune or polygenic complexity [104,105].

Humanized mouse systems incorporating human HLA alleles or immune components represent an intermediate translational strategy. While these models enhance immunological relevance, they remain constrained by incomplete recapitulation of systemic human physiology and long-term disease heterogeneity [106].

#### 4.2.4. Integrating Experimental Models into the Genetic Continuum Framework

Taken together, monogenic and polygenic experimental systems represent distinct points along the genetic continuum of diabetes. Monogenic models provide high-resolution insight into isolated molecular pathways, effectively amplifying specific components of β-cell dysfunction or metabolic stress. Polygenic models incorporate immune or metabolic complexity but remain constrained by limited genetic diversity and controlled environmental conditions. Neither category fully reproduces the heterogeneous, environmentally modulated, and temporally dynamic nature of human diabetes.

Importantly, both model types converge on shared biological endpoints, β-cell vulnerability, impaired stress adaptation, and dysregulated immune–metabolic cross-talk, thereby reinforcing the continuum-based framework proposed in this review. Experimental models should therefore be interpreted not as direct replicas of human disease, but as functional abstractions that clarify discrete mechanistic nodes within a broader and more complex biological network. When integrated with human genomic, clinical, and multi-omics data, these systems remain indispensable for understanding the convergent pathways unifying monogenic and polygenic diabetes.

## 5. Translational Perspectives

### 5.1. Genetic Testing and Diagnosis

Advances in next-generation sequencing (NGS) technologies have fundamentally transformed the diagnostic approach to diabetes of uncertain etiology, enabling comprehensive and cost-effective analyses of genes associated with monogenic diabetes within a single assay [107,108]. Targeted gene panels, whole-exome sequencing, and, in selected cases, whole-genome sequencing have substantially increased diagnostic yield, particularly in individuals with young-onset diabetes, preserved C-peptide secretion, negative islet autoantibodies, and atypical clinical features [107,108].

Accurate genetic diagnosis has immediate clinical consequences. Identification of a monogenic etiology prevents misclassification as type 1 or type 2 diabetes, avoids unnecessary or inappropriate insulin therapy, and supports individualized treatment strategies tailored to the underlying molecular defect [108,109]. In addition, genetic diagnosis enables cascade testing of family members, facilitates reproductive counseling [110], and informs long-term surveillance for extra-pancreatic manifestations in syndromic forms of diabetes. From a health system perspective, early molecular diagnosis may reduce cumulative healthcare costs by optimizing therapy and minimizing complications associated with suboptimal disease management.

Despite these advantages, genetic testing remains underutilized in routine clinical practice. Barriers include limited clinician awareness, uncertainty regarding patient selection, variability in access to genetic services, and challenges in interpreting variants of uncertain significance. Addressing these obstacles will be essential for integrating genetic testing into standardized diagnostic algorithms and for ensuring equitable access to precision diabetology [107,108,109,111].

### 5.2. Therapeutic Implications

Monogenic diabetes provides a clear and compelling paradigm for genotype-guided therapy, in which molecular diagnosis directly informs treatment choice. The most established examples include sulfonylurea sensitivity in patients with *HNF1A*- or *HNF4A*-related diabetes and the successful replacement of insulin with oral sulfonylureas in many individuals with KATP-channel-related neonatal diabetes [108,109]. These cases illustrate how understanding disease mechanism can dramatically alter therapeutic strategy and patient quality of life.

Beyond rare monogenic forms, insights gained from genetic studies increasingly inform therapeutic decision-making in polygenic diabetes. Genetic variation may influence drug response, treatment durability, and susceptibility to adverse effects, contributing to interindividual variability in therapeutic outcomes [112,113]. For example, variants affecting β-cell function, incretin signaling, or insulin sensitivity may modulate response to glucose-lowering agents such as sulfonylureas, GLP-1 receptor agonists, or insulin secretagogues [114]. Although these associations are not yet sufficiently robust to guide routine clinical decisions, they underscore the potential of pharmacogenomics to refine treatment selection in the future.

Importantly, genotype-guided therapy in type 2 diabetes remains an aspirational goal rather than standard practice. Current treatment algorithms are still largely phenotype-driven, relying on clinical features such as body weight, glycemic control, and comorbidities. Nevertheless, accumulating evidence supports the future integration of genetic information into personalized treatment frameworks, particularly as subclassification approaches and pharmacogenomic datasets mature [28,111,113]. The challenge lies in translating probabilistic genetic information into actionable clinical guidance without oversimplification or therapeutic inertia.

### 5.3. Nutrigenetics and Precision Prevention

Nutrigenetics explores how genetic variation influences individual metabolic responses to dietary composition and lifestyle interventions, offering a potential pathway toward personalized diabetes prevention and management [115,116]. Variants associated with obesity, insulin resistance, and β-cell dysfunction have been shown to modulate glycemic responses to macronutrient intake, caloric restriction, and weight-loss strategies, highlighting the biological basis for interindividual variability in dietary efficacy [115,116].

In the context of diabetes prevention, integrating genetic risk with lifestyle modification may enhance the effectiveness of interventions, particularly among individuals identified as high risk through polygenic profiling. For example, individuals with a high genetic burden for β-cell dysfunction may benefit disproportionately from early interventions that reduce metabolic stress, whereas those with predominant insulin resistance may respond more favorably to targeted weight-loss strategies [111,116]. However, the clinical utility of nutrigenetic approaches remains constrained by modest effect sizes, heterogeneity across populations, and limited prospective validation.

At present, nutrigenetics should be viewed as a complementary tool rather than a standalone solution [117]. Its greatest value may lie in motivating adherence to lifestyle interventions and in refining population-level prevention strategies when combined with clinical, metabolic, and behavioral data. As evidence accumulates and analytical frameworks improve, nutrigenetics may contribute to a more nuanced and individualized approach to diabetes prevention within a broader precision medicine paradigm [111,116].

## 6. Future Directions

The evolving genetic landscape of diabetes highlights both the power and the limitations of current molecular approaches. While genomics has substantially improved our understanding of inherited susceptibility and disease mechanisms, genetic information alone provides a largely static view of risk and does not capture the dynamic processes that drive disease onset, progression, and response to therapy [118]. Future advances in diabetology will therefore require integrative strategies capable of linking genetic predisposition with real-time molecular readouts of metabolic and cellular stress.

### 6.1. From Inherited Risk to Dynamic Disease Monitoring

A central challenge in diabetes management lies in monitoring the transition from genetic susceptibility to overt β-cell dysfunction and clinical disease. PRS and monogenic diagnoses identify individuals at increased risk or with specific molecular defects, but they do not reflect ongoing β-cell injury, inflammatory activity, or metabolic adaptation [19,25]. Longitudinal biomarkers capable of capturing disease activity over time are needed to complement genetic stratification and to inform personalized intervention strategies.

In this context, the integration of multi-omics approaches represents a promising direction [119]. Transcriptomic, epigenomic, proteomic, and metabolomic profiling can provide functional insights into how genetic variation is translated into cellular phenotypes [120]. When applied longitudinally, these approaches may enable the identification of molecular signatures associated with β-cell stress, declining insulin secretory capacity, or response to therapeutic interventions, thereby refining disease subclassification beyond static clinical labels.

### 6.2. Liquid Biopsy as an Emerging Tool in Precision Diabetology

Among emerging molecular approaches, liquid biopsy has attracted increasing interest as a minimally invasive method for accessing disease-relevant biological information [121]. Initially developed in oncology [122,123], liquid biopsy is now being explored in metabolic diseases as a means to assess tissue-specific stress, intercellular communication, and systemic metabolic remodeling when direct tissue sampling is impractical or impossible [121].

Liquid biopsy encompasses a heterogeneous set of circulating biomarkers, including cell-free nucleic acids, extracellular vesicles, and metabolites, each of which may provide complementary information about diabetes pathophysiology [121,124]. Circulating cell-free DNA and RNA may reflect tissue injury or altered gene expression patterns, while extracellular vesicles can convey signals between metabolically active organs such as pancreatic islets, liver, adipose tissue, and skeletal muscle [121,125,126]. Metabolomic profiling offers a functional snapshot of systemic metabolic state and may reveal early perturbations preceding overt hyperglycemia [127].

In type 2 diabetes, liquid biopsy approaches hold potential for early detection, monitoring of disease progression, and risk stratification for complications, particularly in individuals with ambiguous phenotypes or discordant clinical markers. When combined with genetic information, liquid biopsy-derived markers could help distinguish individuals with predominant β-cell failure from those driven primarily by insulin resistance, thereby informing more tailored preventive or therapeutic strategies.

### 6.3. Challenges and Translational Considerations

Despite its promise, the application of liquid biopsy in diabetes faces significant challenges. These include limited tissue specificity of circulating markers, lack of standardized analytical pipelines, variability in pre-analytical and post-analytical procedures, and uncertainty regarding clinical thresholds and longitudinal interpretation. Moreover, the integration of liquid biopsy data with genetic and clinical information will require robust computational frameworks and careful validation in diverse populations.

Ethical and practical considerations must also be addressed. As with genetic testing, issues related to data interpretation, patient communication, cost-effectiveness, and equitable access are central to responsible implementation. Importantly, future research should prioritize prospective studies designed to assess whether liquid-biopsy-guided strategies improve clinical outcomes beyond existing standards of care.

### 6.4. Toward Integrated Precision Diabetes Care

Looking forward, the convergence of genetics, multi-omics profiling, and liquid biopsy offers an opportunity to move beyond static classification systems toward a more dynamic and biologically grounded model of diabetes care. In such a framework, inherited genetic risk would define susceptibility, while circulating molecular markers would track disease activity and therapeutic response over time. Although significant methodological and translational hurdles remain, this integrative approach has the potential to transform diabetes management from reactive glycemic control to proactive, mechanism-driven precision care (Table 3).

## 7. Conclusions

Diabetes mellitus is increasingly recognized as a biologically heterogeneous disorder that extends beyond traditional phenotype-based classifications. Advances in human genetics have revealed that monogenic and polygenic forms of diabetes are not discrete entities, but rather represent points along a continuum of genetic architectures that converge on shared molecular pathways governing β-cell identity, function, and survival. Rare monogenic disorders provide high-resolution insight into these pathways, while common polygenic forms reflect their cumulative and environmentally modulated disruption.

By integrating evidence from monogenic diabetes, genome-wide association studies, and emerging translational research, this review highlights how diverse genetic perturbations ultimately impair a limited set of core biological processes, including transcriptional regulation, glucose sensing, mitochondrial metabolism, and cellular stress responses. Recognition of these shared mechanisms challenges rigid diagnostic labels and supports a shift toward a more mechanistic, molecularly informed framework for understanding diabetes pathophysiology.

Importantly, genetic insights are already reshaping clinical practice in selected contexts, enabling precise diagnosis, targeted therapy, and improved risk stratification. At the same time, the limitations of static genetic information underscore the need for complementary approaches capable of capturing dynamic disease processes. The integration of genetics with functional molecular profiling and longitudinal biomarkers offers a promising path toward more refined disease subclassification and personalized management.

In conclusion, genetics provides a unifying framework for understanding the diversity of diabetes, bridging rare and common forms through shared biological vulnerability. As molecular tools continue to evolve, their thoughtful integration into clinical care holds the potential to transform diabetes management from a predominantly phenotype-driven approach to a truly precision-based discipline, grounded in mechanism, biology, and individual risk.

## Figures and Tables

**Table 1:** Monogenic diabetes: genes, mechanisms, and clinical implications. MODY Maturity-Onset Diabetes of the Young, NDM Neonatal Diabetes Mellitus, *HNF1A* Hepatocyte Nuclear Factor 1 Alpha, *HNF4A* Hepatocyte Nuclear Factor 4 Alpha, *HNF1B* Hepatocyte Nuclear Factor 1 Beta, *GCK* Glucokinase, *PDX1* Pancreatic and Duodenal Homeobox 1, NEUROD1 Neuronal Differentiation Factor 1, *KLF11* Kruppel-Like Factor 11, CEL Carboxyl Ester Lipase, *INS* Insulin, *KCNJ11* Potassium Inwardly Rectifying Channel Subfamily J Member 11, ABCC8 ATP Binding Cassette Subfamily C Member 8, *EIF2AK3* Eukaryotic Translation Initiation Factor 2 Alpha Kinase 3, KATP channel ATP-sensitive potassium channel, ER stress Endoplasmic Reticulum stress.

Gene	Diabetes Subtype	Primary Mechanism	Typical Phenotype	Therapeutic Implication
*HNF1A*	MODY3	Transcriptional dysregulation	Progressive insulin secretory failure	High sulfonylurea sensitivity
*HNF4A*	MODY1	Transcriptional regulation	Early-onset diabetes ± macrosomia	Sulfonylurea responsiveness
*HNF1B*	MODY5	Developmental defect	Diabetes + renal/urogenital anomalies	Often insulin required
*GCK*	MODY2	Impaired glucose sensing	Mild stable fasting hyperglycemia	No treatment (except pregnancy)
*PDX1*	MODY	β-cell development	Variable insulin deficiency	Often insulin
*INS*	MODY/NDM	Proinsulin misfolding	Variable, stress-related β-cell failure	Insulin
*KCNJ11*	NDM	KATP channel dysfunction	Neonatal diabetes ± neuro features	Sulfonylureas
*ABCC8*	NDM/MODY-like	KATP channel dysfunction	Neonatal diabetes	Sulfonylureas
*EIF2AK3*	Syndromic NDM	ER stress	Diabetes + multisystem disease	Insulin

**Table 2:** Genetic architecture and biological pathways in polygenic diabetes. HLA Human Leukocyte Antigen, TCF7L2 Transcription Factor 7 Like 2, PRS Polygenic Risk Scores.

Feature	Type 1 Diabetes	Type 2 Diabetes
Genetic architecture	Polygenic, immune-dominated	Polygenic, regulatory
Major loci	HLA class II	TCF7L2, multiple regulatory loci
Primary biological target	Immune tolerance	β-cell function and survival
Role of environment	Modifying	Amplifying
β-cell involvement	Immune-mediated destruction	Genetically vulnerable β-cells
Clinical heterogeneity	Age of onset, progression	Onset, insulin resistance vs. failure
Role of PRS	Risk prediction, stratification	Early-onset and subtype identification

**Table 3:** Molecular tools in precision diabetology: current status and future potential. PRS Polygenic Risk Scores, cfDNA Cell-free DNA, EVs Extracellular Vesicles.

Tool	What It Measures	Current Clinical Use	Future Potential
Monogenic testing	Single-gene defects	Established	Expanded screening
PRS	Inherited risk	Research/limited	Risk stratification
Transcriptomics	Gene expression	Research	Subclassification
Metabolomics	Functional metabolism	Research	Disease monitoring
Liquid biopsy	cfDNA, EVs, metabolites	Experimental	Dynamic disease tracking

## Data Availability

No dataset was generated for the publication of this article.
